# The effect of systemic inflammation on human brain barrier function

**DOI:** 10.1016/j.bbi.2016.10.020

**Published:** 2017-05

**Authors:** Elliot Elwood, Zhi Lim, Hammad Naveed, Ian Galea

**Affiliations:** Clinical Neurosciences, Clinical & Experimental Sciences, Faculty of Medicine, University of Southampton, Southampton, UK

**Keywords:** Blood-brain barrier, Systemic, Inflammation, Infection, Cerebrospinal fluid, Albumin

## Abstract

The blood-brain barrier (BBB) plays an important role in the clinical expression of neuropsychiatric symptoms during systemic illness in health and neurological disease. Evidence from *in vitro* and preclinical *in vivo* studies indicate that systemic inflammation impairs blood-brain barrier function. In order to investigate this hypothesis, we evaluated the association between systemic inflammatory markers (leucocytes, erythrocyte sedimentation rate and C-reactive protein) and BBB function (cerebrospinal fluid/serum albumin ratio) in 1273 consecutive lumbar punctures. In the absence of cerebrospinal fluid (CSF) abnormality, systemic inflammation did not affect the CSF/serum albumin ratio. When CSF abnormality was present, systemic inflammation significantly predicted the CSF/serum albumin ratio. Amongst the systemic inflammatory markers, C-reactive protein was the predominant driver of this effect. Temporal analysis in this association study suggested causality. In conclusion, the diseased BBB has an increased susceptibility to systemic inflammation.

## Highlights

• Systemic inflammation impairs blood-brain barrier (BBB) in preclinical models.
• In humans, BBB function was assessed by the CSF/serum albumin ratio (CSAR).
• Systemic inflammation did not affect CSAR in the non-inflamed brain.
• Systemic inflammation increased CSAR in the presence of brain inflammation.
• The diseased BBB has an increased susceptibility to systemic inflammation.

## 1 Introduction

The blood-brain barrier (BBB) is a highly regulated interface between the brain and the rest of the body (Abbott et al., 2010). BBB permeability is an important aspect of several important neurological conditions such as multiple sclerosis and Alzheimer’s disease (Varatharaj and Galea, 2016). Recent compelling evidence suggests that BBB integrity is an important player in the expression of neuropsychiatric manifestations of circulating anti-NMDA antibodies (Hammer et al., 2014). It is interesting to note that preceding systemic infections are very common in neuronal surface antibody-mediated encephalitis (Irani et al., 2010); the aetiology of these prodromal infections are very varied, arguing against molecular mimicry, and perhaps more supportive of a generic effect of systemic infection on the BBB.

A number of *in vitro* and *in vivo* preclinical studies have shown that inflammatory challenge results in an increase in BBB permeability. This effect appears to be a feature of the BBB, unrelated to the type of inflammatory trigger since it has been observed in a wide variety of experimental settings including lipopolysaccharide, poly I:C, bacteria, viruses, chemically-induced inflammation, anaphylaxis and cytokines (Varatharaj and Galea, 2016). The main mechanisms underlying this phenomenon are tight junction changes and increased vesicular transport, but re-induction of fenestrae, endothelial cell damage, denudation of the glycocalyx, degradation of the glia limitans and astrocyte changes also play a role (Varatharaj and Galea, 2016).

While the effect of inflammatory challenges on human BBB permeability has been demonstrated *in vitro* using human brain microvessel endothelial cells (Varatharaj and Galea, 2016), the relevance of this large body of preclinical literature to the *in vivo* human situation remains to be shown. In this study, we set out to study the effect of systemic inflammation on human BBB permeability *in vivo* by examining the association between a panel of systemic inflammatory markers and the CSF/serum albumin quotient in 1273 consecutive unselected lumbar punctures. The CSF/serum albumin ratio (Q_alb_) is a widely accepted indicator of blood–CSF barrier function (Thompson, 2005). Since albumin is not synthesised in the brain, the ratio of CSF to serum albumin concentration is a quotient representing the fraction of serum albumin diffusing into the CSF, independent of serum concentration. Changes in serum albumin do not occur rapidly; hence CSF albumin can be assumed to be in constant equilibrium with serum as a function of BBB permeability. Immunoglobulins and cytokines are not suited for this type of study since they may be secreted intrathecally by blood-derived cells transmigrating into the brain. The effectiveness of Q_alb_ for measurement of BBB function has been demonstrated by studies with radiolabelled albumin (Tourtellotte et al., 1980).

## 2 Methods and materials

### 2.1 Data collection

**Fig. 1:**
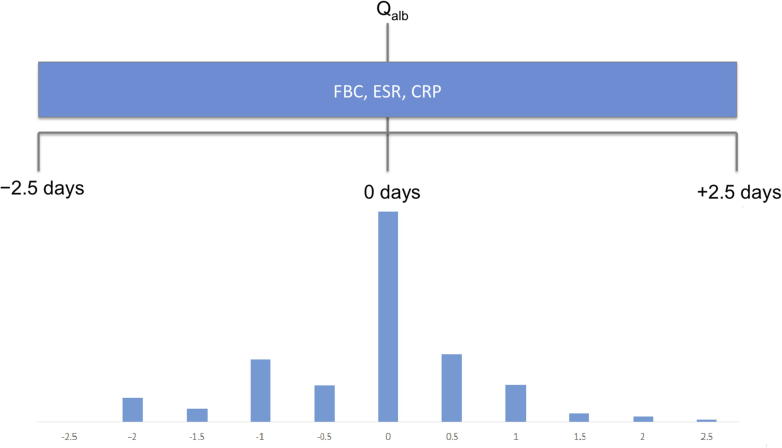
Study design. FBC: full blood count, ESR: erythrocyte sedimentation rate, CRP: C-reactive protein.

Data was collected by retrospective review of the medical records of 1273 individuals having lumbar puncture with Q_alb_ assessment at Southampton General Hospital, Hampshire, UK in a three year period (2011–2013), during a service evaluation, with institutional approval. The white cell count, neutrophil, lymphocyte, erythrocyte sedimentation rate (ESR) and C-reactive protein (CRP) measurements within a five day period centred around the lumbar puncture were recorded (Fig. 1).

We additionally collected data on: age, CSF total protein, CSF glucose, CSF red and white blood cells, and oligoclonal bands. Cases were excluded if <16 years of age and/or had a CSF red blood cell (RBC) count >127 cells per microlitre. Since CSF total protein concentration rises by 1 mg/dl for every 100 red blood cells/μL that enter the CSF during traumatic lumbar puncture (Blakeley and Irani, 2009), the CSF RBC count threshold was determined by calculating the maximum CSF RBC count which did not change any of the Q_alb_ values in the dataset.

CSF and blood were collected in sterile polypropylene tubes (Sterilin, Newport, Gwent, UK) and Vacutainers (Becton Dickinson, Plymouth, UK) respectively. CSF volume was not available. Samples were analysed on the same day, except for isoelectric focussing in which case samples were kept at 4 °C and batch analysed within one week. Blood counts were performed on a Sysmex XE-2100 automated hematology system. ESR was performed on a Vitech Starrsed system using the Westergren sedimentation method. CRP, albumin, protein and glucose were assayed on a Beckman Coulter AU5800 automated system. CSF cell counts were performed manually using a modified Fuchs-Rosenthal haemocytometer. Oligoclonal band assessment was performed manually using isoelectric focussing on CSF/serum pairs.

### 2.2 Data preparation

Data preparation was performed in Excel v14. Cases were identified as having normal findings if the following conditions were met: CSF total protein <500 mg/L, CSF glucose >2/3 serum glucose, white blood cells ⩽5 cells/μL, polymorphs were absent, and there was no evidence of intrathecal synthesis of oligoclonal immunoglobulin G. Single systemic inflammatory variables included total leucocytes, neutrophils, lymphocytes, ESR and CRP; a composite variable (Inf_Blood_) integrating all these indices was created to reflect systemic inflammation. To reflect central nervous system inflammation, the variable Inf_CSF_ was derived from the CSF white cell count. The derivation of Inf_Blood_ and Inf_CSF_ is detailed in Supplementary Methods.

### 2.3 Statistical analysis

Statistical analysis was performed in SPSS v22. Since data was non-parametric, Mann-Whitney test was used for group comparisons. General linear model was used for analysis of covariance. Q_alb_, Inf_CSF_, and systemic inflammatory variables were logarithmically transformed. Multivariate linear regression was used to examine the association of systemic inflammatory markers with Q_alb_. A significant difference from the null hypothesis was assumed at p < 0.05.

## 3 Results

### 3.1 Characteristics of cases

Out of a total of 1273 lumbar punctures, 829 cases were identified with a full CSF analysis including Q_alb_ and at least one systemic inflammatory variable result available within the 5 day period centred around the lumbar puncture. The characteristics of this study population are shown in Table 1, Table 2. Median CRP was 4 mg/L, with an interquartile range of 14 mg/L, and mean CRP was 20 mg/L, with a standard deviation of 43 mg/L, representing predominantly mild-to-moderate systemic inflammation, rather than septic conditions.

### 3.2 Cases with normal CSF

**Fig. 2:**
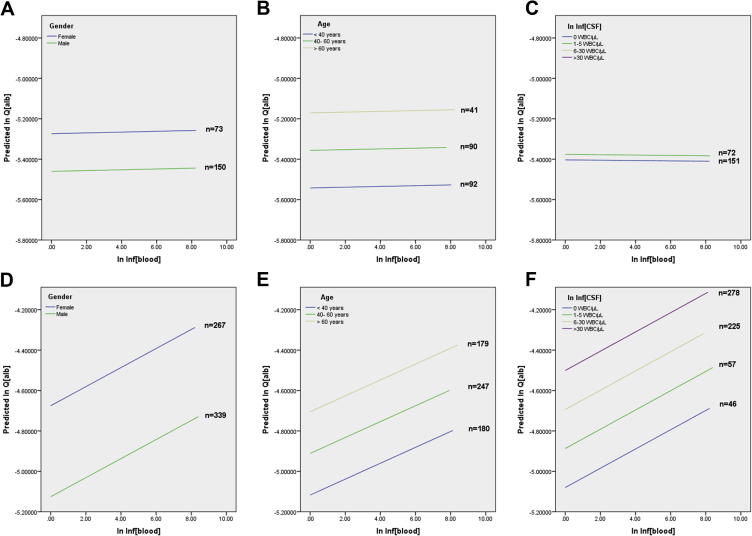
Relationship between systemic inflammation and blood-brain barrier permeability in the presence of normal (A,B,C) and abnormal (D,E,F) CSF. Regression lines of Inf_Blood_ versus Q_alb_ across sex (A,D) and quantiles of age (B,E) and Inf_CSF_ (C,F). To generate this Figure, Q_alb_ was regressed against Inf_Blood_ across categories of sex (A,D), age (B,E) and Inf_CSF_ (C,F).

In cases with normal CSF findings (n = 223), age, sex and Inf_Blood_ were entered as predictors in a multiple linear regression model with Q_alb_ as dependent variable (Table 3, Fig. 2A,B,C). Age was a significant predictor, sex was a borderline predictor, but none of the systemic inflammatory variables (composite or single) significantly predicted Q_alb_. Hence in individuals with a quiescent CSF, systemic inflammation was not associated with blood-CSF barrier permeability to albumin.

### 3.3 Cases with abnormal CSF

Q_alb_ was significantly higher in cases with abnormal CSF versus those with normal CSF (n = 606 and 223, medians of 0.007 and 0.005 respectively, p < 10^−25^), and this effect was still significant in an analysis of covariance with age and sex (p < 10^−8^). This was expected since several neurological diseases affect BBB permeability (Rosenberg, 2012). This finding confirmed the necessity to include CSF abnormality as a covariate while examining the effect of systemic inflammation on Q_alb_ in the presence of CSF abnormality. For this purpose, the CSF cell count was used as an indicator of CSF abnormality, in the form of the normalized variable Inf_CSF_.

Age, sex, Inf_Blood_ and Inf_CSF_ were entered as predictors in a multiple regression model with Q_alb_ as dependent variable. All four variables were significant predictors of Q_alb_ (Table 3 and Fig. 2D,E,F).

**Fig. 3:**
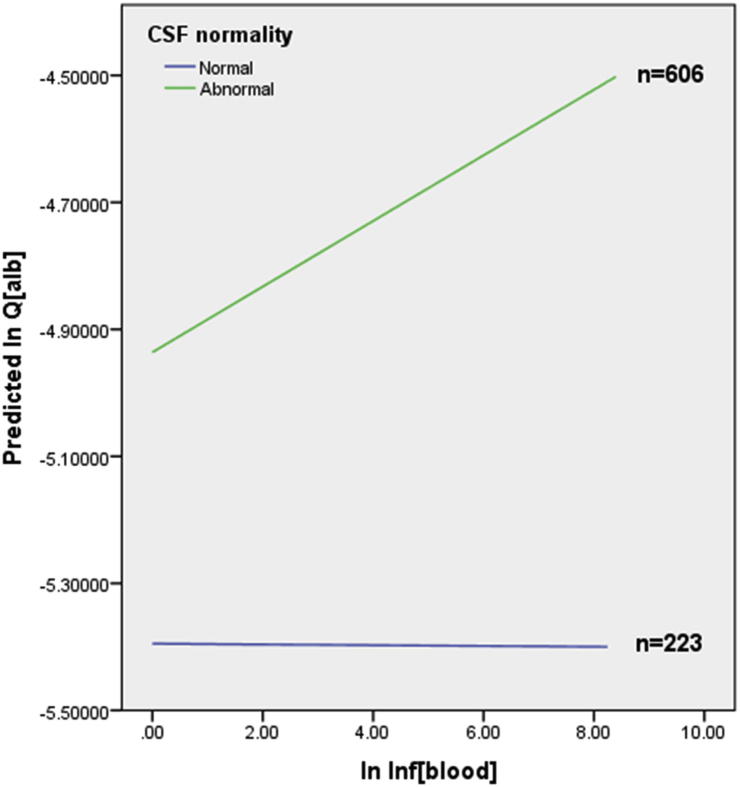
Regression lines of Inf_Blood_ versus Q_alb_ showing relationship between systemic inflammation and blood-brain barrier permeability in cases with normal and abnormal CSF. To generate this Figure, Q_alb_ was regressed against Inf_Blood_, CSF normality and an interaction between Inf_Blood_ and CSF normality.

Inf_Blood_ predicted Q_alb_ in the presence of CSF abnormality, but not when CSF was normal. Hence the effect of systemic inflammation on Q_alb_ was different between cases with normal and abnormal CSF; an interaction between Inf_Blood_ and CSF normality was significant (Fig. 3, p = 0.009). In view of the known interplay between systemic and brain inflammation (Combrinck et al., 2002, Cunningham et al., 2005), an interaction term between Inf_Blood_ and Inf_CSF_ was explored as a second block after age, sex, Inf_Blood_ and Inf_CSF_ across all cases; this was significant (p < 0.03). In cases with abnormal CSF only, an interaction between Inf_Blood_ and Inf_CSF_ was examined in the regression analysis as a second block after age, sex, Inf_Blood_ and Inf_CSF_; a trend was observed but the interaction was not significant (p = 0.08).

When individual systemic inflammatory markers were examined, high CRP and neutrophils exhibited the same relationship with Inf_CSF_ and Q_alb_, but CRP exerted the highest influence on BBB permeability (beta = 0.128 and 0.077, p = 0.01 and 0.04 for CRP and neutrophils respectively). Hence CRP was the most sensitive systemic inflammatory marker impacting on BBB permeability.

### 3.4 Temporal analysis

In order to investigate whether the relationship between systemic inflammation and Q_alb_ was cause or effect, a temporal analysis was undertaken. The multiple linear regression was re-run in cases with abnormal systemic inflammatory markers (Inf_Blood_, CRP or neutrophils) before the LP (n = 59), and in cases with normal systemic inflammatory markers before the LP but abnormal markers at or after the LP (n = 29). High systemic inflammatory markers (Inf_Blood_, CRP or neutrophils) were only predictive of an increased Q_alb_ when these systemic abnormalities were present before the LP (beta = 0.366, p = 0.001 for Inf_Blood_; beta = 0.234, p = 0.046 for CRP; beta = 0.314, p = 0.003 for neutrophils). This result is in keeping with a causative relationship of systemic inflammation on BBB breakdown, in the presence of intrathecal inflammation.

## 4 Discussion

This study is the first to explore the association between systemic inflammation and BBB function in humans, demonstrating a vulnerability of the BBB in the diseased brain to the effect of systemic inflammation. While systemic inflammation did not affect the blood-CSF barrier in individuals with quiescent CSF, systemic inflammation significantly influenced blood-CSF barrier permeability in the presence of CSF abnormality. It is possible that the techniques used in this study were not sufficiently sensitive to detect an effect of systemic inflammation on BBB function in the healthy brain. In particular Q_alb_ may underestimate, or fail to detect, changes in BBB permeability to molecules smaller than albumin. Despite this limitation, this study has clearly demonstrated that the BBB in the diseased brain is more susceptible to systemic inflammation compared to the healthy brain. The BBB alterations which occur as a consequence of neuropathology (Rosenberg, 2012) may be responsible for this phenomenon.

The findings in this study are similar to observations in preclinical studies. In healthy animals, BBB permeability was significantly affected when septic doses, but not lower doses, of lipopolysaccharide were used (Banks et al., 2015, Jaeger et al., 2009). In contrast, a more widespread increase in BBB permeability is seen after LPS challenge in animals with neuropathology such as experimental autoimmune encephalomyelitis, in brain (Serres et al., 2009) and spinal cord (Lopez-Ramirez et al., 2014). Increased BBB vulnerability to systemic inflammation was seen in an Alzheimer disease mouse model, compared to wild-type mice (Takeda et al., 2013). Likewise, the BBB in ischaemic stroke is more susceptible to systemic inflammation (Denes et al., 2011).

Systemic inflammation affects the course of neurological disease. Systemic infection causes accelerated cognitive decline in patients with AD (Holmes et al., 2009) and impairs outcome in stroke patients (Smith et al., 2004). Experimentally-induced systemic inflammation in preclinical models of AD (Cunningham et al., 2005) and stroke (Denes et al., 2011) has confirmed a causative association. It is possible that the effect of systemic inflammation on the BBB may be one of the mechanisms underlying the worsening of neurological disease with systemic inflammation. In support of this, experimental disruption of the BBB results in neurological toxicity (Tomkins et al., 2007) and experimental manoeuvres which strengthen the BBB result in improvement of pathology (Hong et al., 2015, Podjaski et al., 2015). BBB alterations induced by systemic inflammation in AD favour increased amyloid deposition in the brain (Jaeger et al., 2009).

Delirium is commonly caused by systemic infection (George et al., 1997) or critical illness (Ouimet et al., 2007), conditions associated with systemic inflammation. Elevated levels of serum S100B, a potential biomarker of BBB disruption (Blyth et al., 2009), have been observed during delirium in Alzheimer’s disease (van Munster et al., 2010) and critical illness (Hughes et al., 2016). Since systemic inflammation affects the BBB, it is possible that increased BBB permeability is a mediator of delirium in conditions such as Alzheimer’s disease and critical illness.

Both systemic infections and BBB permeability have been linked to clinical outcome. For example in experimental stroke, systemic IL1β challenge results in matrix metalloproteinase 9-induced BBB disruption, larger infarct size and poorer neurological outcome (McColl et al., 2008). In multiple sclerosis, systemic infections occur in conjunction with gadolinium enhancement and clinical relapses (Correale et al., 2006). Further work is needed to determine the importance of the *systemic infection → BBB breakdown → clinical outcome* mechanistic pathway in neurological patients. Prevention and/or aggressive treatment of systemic infections, and the use of agents which protect against or reverse systemic inflammation-induced increase in BBB permeability (McColl et al., 2008, Yang et al., 2015) would be potential therapeutic avenues.

Future work is needed to address limitations of this study. Here, we have not systematically determined the precise reason for systemic raised inflammatory markers or abnormal CSF. Although preclinical studies have shown that the effect of systemic inflammation on BBB permeability occurs across a broad range of systemic inflammatory stimuli and neuropathologies (Varatharaj and Galea, 2016), future studies may explore which types of inflammatory stimuli and neuropathologies are combinatorially most conducive to an interaction between the periphery and the brain. Since this was a retrospective study, information on mental state or illness behaviour was not available. The use of Q_alb_ to evaluate BBB function has its own limitations, particularly the possibility of confounding by CSF bulk flow (Reiber, 1994, Thompson, 2005), which can be influenced by systemic inflammation (Erickson et al., 2012). Finally since Q_alb_ represents the blood-CSF barrier, which is only one of the barriers in the central nervous system (Abbott et al., 2010), other methods of studying human BBB function *in vivo* such as dynamic contrast-enhanced magnetic resonance imaging (DCE-MRI) or circulating biomarkers of BBB integrity will be useful. Although this study provided some evidence in support of causation, prospective and/or experimental human studies would provide concrete proof that systemic inflammation affects BBB function.

## Conflict of interest statement

All authors declare that there is no conflict of interest.

## Figures and Tables

**Table 1:** Demographics.

Characteristic	
Number	829
Age (years, ±SD)	49 ± 17
Sex (% male)	41%
Timing of blood sample to lumbar puncture (%)	
Before LP	38
At LP	36
After LP	26
Final neurological diagnosis (%)	
Inflammatory	31
Idiopathic	12
Degenerative	6
Vascular	6
Infectious	4
Neoplastic	4
Metabolic	1
Miscellaneous	5
Multiple neurological diagnoses	4
No neurological diagnosis	27

**Table 2:** Laboratory parameters.

Laboratory parameter (n with data)	% normal			Median (IQR)	Mean (SD)	Reference range
	Normal	Abnormal	Not available			
CSF normality (829)	27	73				
Total white cell count (798)	82	14	4	7.5 (3.7)	8.3 (4.2)	4–11 × 10^9^/L
Neutrophil count (797)	80	17	3	4.6 (2.9)	5.5 (3.2)	2–7.5 × 10^9^/L
Lymphocyte count (797)	70	26	4	1.9 (0.9)	2 (1.3)	1.5–4 × 10^9^/L
Erythrocyte sedimentation rate (297)	24	12	64	10 (19)	19 (23)	1–10 mm/h
C-reactive protein (507)	38	23	39	4 (14)	20 (43)	0–7.5 mg/L
Inf_Blood_ (829)	66	34				

**Table 3:** Multiple linear regression of age, sex, Inf_CSF_ and Inf_Blood_ against Q_alb_.

Variable	Unstandardized Coefficient		Standardized Coefficient	p
	B	Std. Error	Beta	
*Cases with normal CSF*				
Age	0.008	0.002	0.324	<0.01∗
Gender	−0.111	0.061	−0.119	0.07
Ln Inf[CSF]	0.009	0.016	0.037	0.57
Ln Inf[Blood]	0.001	0.011	0.003	0.96

*Cases with abnormal CSF*				
Age	0.009	0.002	0.219	<0.01∗
Gender	−0.414	0.054	−0.282	<0.01∗
Ln Inf[CSF]	0.075	0.011	0.250	<0.01∗
Ln Inf[Blood]	0.029	0.010	0.107	<0.01∗

∗ p < 0.05.
