# Supplementary material for: The effect of systemic inflammation on human brain barrier function
Source: Brain Behav Immun. 2017 May;62:35–40. doi: 10.1016/j.bbi.2016.10.020 (PMC5380128; doi:10.1016/j.bbi.2016.10.020)
Supplement: Supplementary data 1 [file mmc1.docx]

**The effect of systemic inflammation on human brain barrier function**

Elliot Elwood, Zhi Lim, Hammad Naveed and Ian Galea

**SUPPLEMENTARY MATERIAL**

**SUPPLEMENTAL METHODS**

**Derivation of the composite variable Inf_Blood_**

The composite variable Inf_Blood_ was derived to enable systemic inflammation to be denoted by a single variable. This *composite variable* integrated *constituent variables*, as follows:

Inf_Blood_: WBC, ESR, CRP

The composite variable was derived using the following methodology:

(1) **Standardization of constituent variables**. Constituent variables were converted to *standardized constituent variables* ie values on a scale that standardised the reference range to 0-100 (see Table 2 in main text for reference ranges for each variable).

Example: for CRP, *standardized CRP* = [(CRP/7.5) × 100] since the CRP reference range was 0-7.5 mg/L

(2) **Mean of standardized constituent variables**. All available *standardized constituent variables* from step (1) were averaged to create a *mean standardized variable* for blood x̄_blood_

x̄_blood_ = [(standardized WBC + standardized ESR + standardized CRP)/3]

(3) **Conversion of *mean standardized variable* to Inf_Blood_**. The *mean standardized variable* for blood (x̄_blood_) from step (2) was multiplied by the number of constituent variables that were abnormal (ie 0, 1, 2 or 3 since there were 3 constituent variables for blood). This was performed in order to:

1. avoid the anomalous situation whereby abnormalities in some but not all *constituent variables* were masked by averaging during step (2)
2. score as 0 all cases with normal *constituent variables*, so that the scores of all these cases were equal and indicative of absence of detectable inflammation.

Examples: if no abnormalities in WBC, ESR and CRP were present: (x̄_Blood_ × 0) = 0; if only CRP was abnormal: Inf_Blood_ = (x̄_Blood_ × 1); if CRP and ESR were abnormal: Inf_Blood_ = (x̄_Blood_ × 2); if all three variables were abnormal: Inf_Blood_ = (x̄_Blood_ × 3).

**Derivation of the variable Inf_CSF_**

**Inf_CSF_** consisted of CSF cell count only, which was standardized as per (1) above.
